# Understanding of Förster Resonance Energy Transfer (FRET) in Ionic Materials

**DOI:** 10.3390/suschem2040031

**Published:** 2021-10-09

**Authors:** Amanda Jalihal, Thuy Le, Samantha Macchi, Hannah Krehbiel, Mujeebat Bashiru, Mavis Forson, Noureen Siraj

**Affiliations:** Department of Chemistry, University of Arkansas at Little Rock, 2801 S. University Ave., Little Rock, AR 72204, USA

**Keywords:** ionic material, FRET, spectator ions, dye sensitized solar cell, near-infrared dyes

## Abstract

Herein, an ionic material (IM) with Förster Resonance Energy Transfer (FRET) characteristics is reported for the first time. The IM is designed by pairing a Nile Blue A cation (NBA^+^) with an anionic near-infrared (NIR) dye, IR820^−^, using a facile ion exchange reaction. These two dyes absorb at different wavelength regions. In addition, NBA^+^ fluorescence emission spectrum overlaps with IR820^−^ absorption spectrum, which is one requirement for the occurrence of the FRET phenomenon. Therefore, the photophysical properties of the IM were studied in detail to investigate the FRET mechanism in IM for potential dye sensitized solar cell (DSSCs) application. Detailed examination of photophysical properties of parent compounds, a mixture of the parent compounds, and the IM revealed that the IM exhibits FRET characteristics, but not the mixture of two dyes. The presence of spectator counterion in the mixture hindered the FRET mechanism while in the IM, both dyes are in close proximity as an ion pair, thus exhibiting FRET. All FRET parameters such as spectral overlap integral, Förster distance, and FRET energy confirm the FRET characteristics of the IM. This article presents a simple synthesis of a compound with FRET properties which can be further used for a variety of applications.

## 1. Introduction

The sun is an obvious source of renewable and clean energy that would provide approximately 300,000 times the required energy for the global population if utilized. Among solar cells, dye-sensitized solar cells (DSSCs) are a type of third-generation photovoltaic cells that utilize dyes to parallel photosynthesis [1-3]. Typically, the production of DSSCs is economical as compared to the first- and second-generation solar cells [4]. However, DSSCs have not achieved the same high efficiencies as other types of solar devices [5]. Therefore, researchers are seeking new photosensitizers [6], electrolytes [7], electrode materials [8], etc., in enhancing the efficiency of DSSCs. This manuscript presents a new class of material that can be utilized as a potential photosensitizer in DSSCs.

Solar radiation consists of approximately 43% visible light, 49% near-infrared (NIR) light, and 7% ultra-violet light [9]. Thus, a dye with a broad absorption spectrum is one of the primary requirements to absorb maximum sunlight, which consequently affects the efficiency of DSSCs [10]. Since a large portion of UV sunlight is absorbed by the ozone layer, the absorbance of a compound in this region is not of significant importance. However, the longer wavelength NIR radiation can be exploited by utilizing NIR dyes (dyes absorbing from 750–1000 nm) in DSSCs [11,12]. Ideally, the sensitizing dye in DSSCs should absorb 80% or more of the solar spectrum (350–900 nm) [13]. The task of finding or synthesizing a single dye with this broad absorbance is quite difficult. However, two or more dyes can be combined which absorb at different wavelength regions of the electromagnetic spectrum to effectively broaden the absorption spectra that subsequently affects DSSC efficiency [14].

Recently, energy relay dyes (ERD) have become an area of interest where a dye that absorbs in a different wavelength region of sunlight can complementarily enhance the absorption characteristics of another photosensitizer to attain broader absorption spectra [10,15]. In the dye combination, one acts as an acceptor while the other dye serves as an ERD that transfers energy to the acceptor and consequently alters the photophysical properties of the acceptor. These two dyes interact via the Förster Resonance Energy Transfer (FRET) mechanism where the ERD transfers energy to the acceptor photosensitizer non-radiatively [10,16].

**Figure 1.**
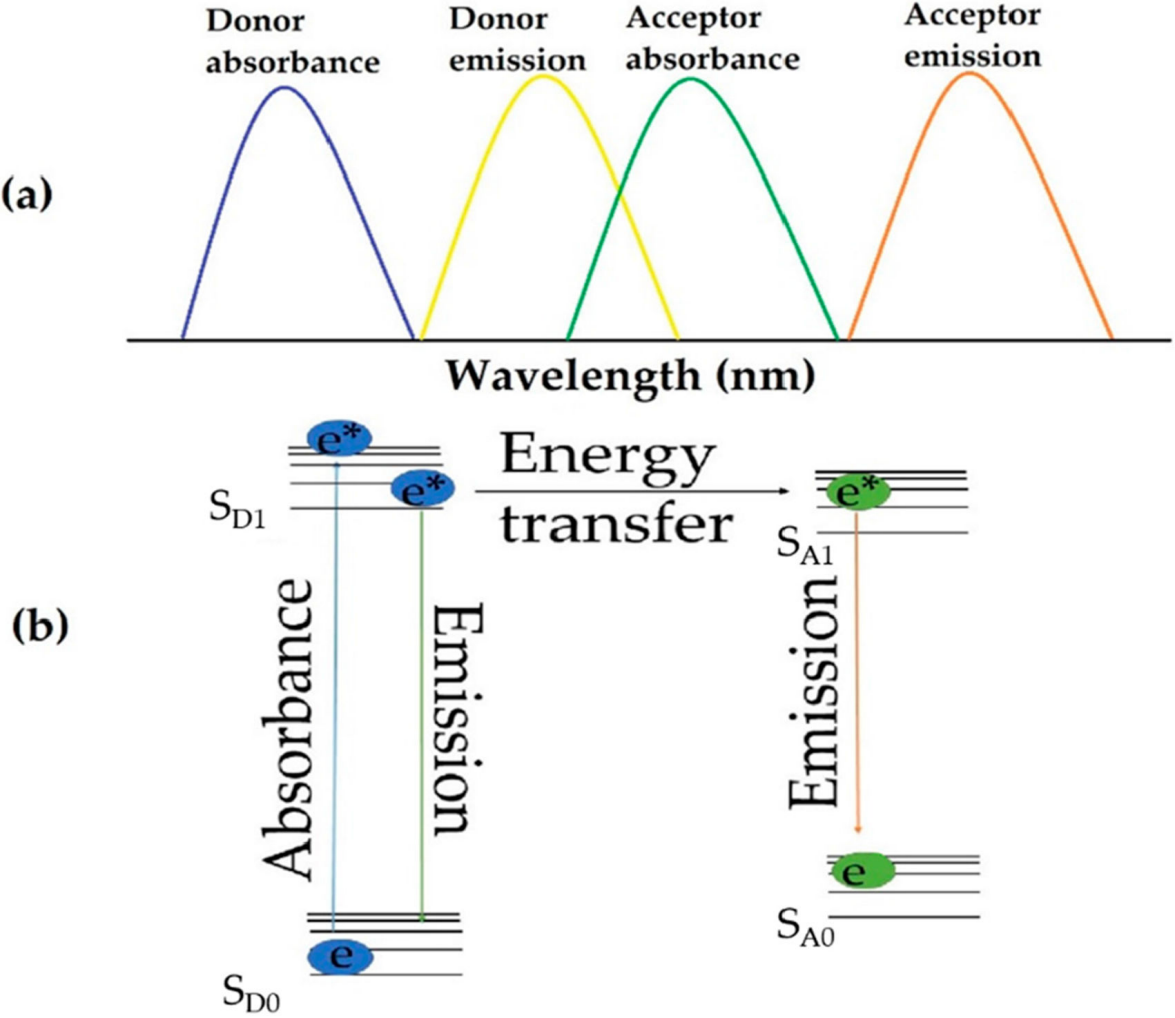
(**a**) Illustration of relationship between donor and acceptor spectra. (**b**) Energy diagram describing energy transfer via FRET mechanism.

FRET is a well-known mechanism of energy transfer that requires a molecule exhibits sufficient spectral overlap between the donor’s emission and the acceptor’s absorbance spectra, and the two species must physically be within 10 nm of each other [17-19]. The energy transfer takes place in the spectral overlap region, as shown in Figure 1a. After absorbing light, the donor is promoted to the excited state and will either radiatively relax by fluorescence or transfer energy to the acceptor non-radiatively. The process is illustrated in Figure 1b. Conventionally, the ERD is simply dissolved in the electrolyte solution of the DSSCs [20], which can lead to quenching of the excited state ERD before reaching the acceptor dye. To acquire high FRET efficiency between ERD dye and acceptor photosensitizer, a new class of compound, ionic materials (IMs), is presented in this work.

IMs are tunable ionic salts that share properties of ionic liquids but are solid at room temperature [21-23]. IMs are ideal for several applications due to their enhanced photo and thermal stability, fluorescence quantum yields, and electrochemical characteristics [10,24,25]. These properties can be further tuned by tailoring counterions using a facile one-step ion exchange method without requiring complex organic synthesis [21]. IMs are advantageous over other organic compounds due to their simple, rapid, and cost-effective synthesis. To the best of our knowledge, this is the first report on FRET-based IMs.

Herein, Nile Blue A (NBA) cation is paired with an anionic NIR dye, IR820, to broaden the absorption spectra of the resulting IM for potential DSSC application. To investigate FRET between donor NBA^+^ and acceptor IR820^−^ dyes, the photophysical properties of Nile Blue A IR820 ([NBA][IR820]) IM, parent dyes, and the mixture of parent dyes are studied in detailed. Parent dyes as well as the mixture of parent dyes contain counterion (sulfate, SO_4_^2−^ and sodium, Na^+^), while in the IM, all counterions have been removed and it contains only two ionic sensitizer dyes (NBA^+^ and IR820^−^). This work also shines light on the effect of spectator ions in FRET mechanism between two ionic dyes.

## 2. Materials and Methods

### 2.1. Materials

2-[2-[2-Cholro-3-[[1,3-dihydro-1,1-dimethyl-3-(4-sulfobutyl)-2*H*-benzo[e]indol-2-ylidene]-ethylidene-1-cyclohexen-1-yl]-ethenyl]-1,1-dimethyl-3-(4-sulfobutyl)-1*H*-benzo [e]indolium hydroxide inner salt (NaIR820), Nile Blue A sulfate (NBA_2_SO_4_), deuterated dimethyl sulfoxide (d-DMSO), and Wilmad 5 mm NMR tubes were purchased from Sigma-Aldrich. Ethanol and dichloromethane (DCM) were purchased from VWR. Alumina cells for thermal stability analysis were purchased from Perkin Elmer. Distilled deionized water of 18.2 MΩ cm was obtained from ultrapure distilled water purifier (ELGA).

### 2.2. Characterization

The identity of the newly synthesized IM was confirmed using a Shimadzu IT-TOF ESI high-resolution mass spectrometer. Fragments were verified by comparing the mass to charge ratio of the ions to the parent dyes. The structure of the IM was verified using proton nuclear magnetic resonance spectroscopy (^1^H-NMR) recorded in d-DMSO with a 300 MHz JEOL ECX instrument. Thermogravimetric analysis (TGA) was preformed using a Mettler Toledo instrument to investigate the thermal stability of the IM. Samples were heated in air at a rate of 10 °C per minute from 25–800 °C.

### 2.3. Absorbance Studies

Samples of parent dyes (NBA_2_SO_4_, NaIR820), the IM ([NBA][IR820]), and the mixture of parent dyes (0.5 NBA_2_SO_4_:1 NaIR820) were prepared in ethanol. Since the IM contains NBA^+^ and IR820^−^ in 1:1 ratio, a mixture was prepared by taking 0.5 mol eq. of NBA_2_SO_4_ with 1 mol eq. of NaIR820 to acquire 1:1 NBA^+^ and IR820^−^ in the mixture. The Shimadzu UV-Vis-NIR spectrophotometer UV-3600 was used to study their absorption characteristics. A 10 mm path length, polished two-sided quartz cuvette (Starna Cells) was used for absorbance measurements against an identical cell filled with the solvent of the sample.

### 2.4. Photoluminescence and Quantum Yield Studies

The fluorescence emission spectra of all samples were recorded at their respective excitation wavelength using the HORIBA Jobin Yvon Nanolog fluorimeter. A 10 mm path length, polished four-sided quartz cuvette (Starna Cells) was used for fluorescence emission measurements. All samples were measured with the same slit width, integration time of 0.1 s, and at right angle geometry. Absolute quantum yield was recorded using a HORIBA integrating sphere at two different excitation wavelengths.

### 2.5. FRET Calculations

All the FRET parameters are calculated to assess the FRET phenomenon in IM. In order to calculate the spectral overlap integral, theoretical Förster distance, and FRET efficiency of the IM, several equations were employed.

First, the spectral overlap integral (*J*(*λ*)) was quantified using Equation (1).

(1) $$J(\lambda )=\frac{\int_{0}^{\infty }\varepsilon (\lambda )f(\lambda )\lambda^{4}d\lambda }{\int_{0}^{\infty }f(\lambda )d\lambda }$$

where *ε*(*λ*) is the molar extinction coefficient (M^−1^cm^−1^) of NaIR820 at overlap wavelength *λ*, and *f*(*λ*) is the normalized fluorescence emission intensity of NBA_2_SO_4_ at overlap wavelength *λ* with NaIR820 when excited at 629 nm [26]. A higher *J*(*λ*) value supports the energy transfer between donor and acceptor via FRET mechanism.

Next, the theoretical Förster distance (*R*_0_) was determined using Equation (2).

(2) $$R_{0}=0.0211(n^{-4}\times k^{2}\times \Phi_{d}\times J{)}^{\frac{1}{6}}$$

where Φ*_d_* is quantum yield of the donor, NBA_2_SO_4_, in the absence of acceptor, NaIR820, *n* is refractive index of the media, *J* is the spectral overlap integral between NBA_2_SO_4_ and NaIR820, and *k*^2^ is the dipole orientation factor [27]. The dipole orientation factor is conventionally estimated as 2/3 [19].

Finally, the FRET efficiency of the IM was calculated using Equation (3).

(3) $$E=1-\frac{F_{da}}{F_{d}}$$

where *F_da_* is integrated fluorescence emission of the donor in the presence of the acceptor and *F_d_* is the integrated fluorescence emission of the donor in the absence of the acceptor [28].

### 2.6. Temperature Dependent Photophysical Characterization

To determine the high operating temperatures of a DSSC effect on the FRET parameters of the IM, temperature studies were performed. A sample was placed in a hot water bath on a Corning PC-420D hot plate. The temperature was measured with a Thomas Scientific Traceable Thermocouple. The absorption and fluorescence emission spectra were recorded at 25, 40, 50, 60, and 70 °C. These resulted spectra were used to calculate all above mentioned FRET parameters at different temperatures.

### 2.7. Synthesis

**Scheme 1.**
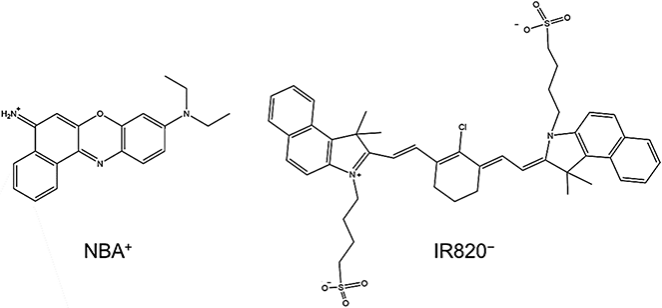
Structures of NBA cation and IR820 anion.

The ionic compound, [NBA][IR820], was synthesized by a previously reported biphasic ion exchange method [29]. Briefly, the NaIR820 parent compound was dissolved in deionized water and the NBA_2_SO_4_ parent compound in DCM. The DCM solution containing the NBA_2_SO_4_ was roughly five times the volume of the aqueous NaIR820 solution and contained a 0.5:1 molee ratio of NBA_2_SO_4_ to NaIR820 parent dyes, which produces 1:1 ratio of NBA^+^ and IR820^−^ in the IM (Equation (4)). The aqueous and organic solutions were combined in a single vessel and stirred for 72 h at room temperature. After agitation, ethanol was added to the biphasic mixture of the aqueous and the organic solvents to pull any remaining aqueous IR820^−^ into the DCM layer. Since ethanol is miscible in both solvents, it was used as an emulsifying agent, along with intense agitation and centrifugation. This process was repeated multiple times until no color in the aqueous layer was observed, thus effectively pushing all aqueous IR820^−^ into the DCM layer. The DCM solution was washed with deionized water multiple times to effectively remove all Na^+^ and SO_4_^2−^ spectator ions. The DCM was removed by evaporation in a reduced pressure environment using rotary evaporator. The remaining IM was then freeze-dried to remove any trace water impurities. The ion exchange reaction for IM synthesis is shown in the balanced chemical Equation (4), and the structures of donor NBA cation and acceptor IR820 anion are illustrated in Scheme 1. The presence of both ions in the newly designed IM was confirmed by mass spectrometry (Figure S1). The observed peaks in positive and negative ion mode were 318.1597 and 827.2946, respectively. This matched well with the calculated molecular weight of NBA^+^ ion (318.1601) and IR820^−^ ion (827.2950). The structure of the IM was confirmed by ^1^H-NMR. Figure S2 presents the ^1^H-NMR spectra which shows the characteristic peaks of hydrogen present in IR820^−^ and NBA^+^ contained in the IM [30,31].


(4) $$2\;{\text{NaIR820}}_{\left(\text{aq}\right)}+{\text{NBA}}_{2}{\text{SO}}_{4\;(\text{DCM})}\overset{\text{stirred}\;72\;\text{hr RT}}{\to }2\;[\text{NBA}][\text{IR820}{]}_{\left(\text{DCM}\right)}+{\text{Na}}_{2}{\text{SO}}_{4\;(\text{aq})}$$

## 3. Results and Discussion

### 3.1. Materials Characterization

The melting point of the [NBA][IR820] compound was recorded to be 240 °C. Then, further characterization of the IM was performed.

Thermal stability of a photosensitizer is a critically important parameter when used for DSSCs application. Most organic compounds degrade at low temperatures [32,33]. This experiment is performed to examine the IM thermal stability in comparison to parent dyes. Thermal stability was investigated for [NBA] [IR820] IM and the respective parent dyes by heating under continuous air flow from 25–800 °C, shown in Figure S3. All three samples gradually degrade similarly until approximately 300 °C, at which point NBA_2_SO_4_ loses significant weight. After 450 °C, [NBA][IR820] IM degrades slightly more than NaIR820, indicating there is no significant change in thermal stability when converting these parent compounds into IM.

### 3.2. Photophysical Properties

Absorption measurements of all parent compounds, the IM, and the mixture of parent compounds are performed in ethanol solvent. As mentioned earlier, the mixture of two parent compounds is prepared by mixing 0.5 mol eq. of NBA_2_SO_4_ with 1 mol. eq. of NaIR820. The resultant mixture of the two parent dyes produces a 1:1 mole ratio of NBA^+^ to IR820^−^ in the mixture. For better comparison, the mole ratio of the two dyes in the mixture was kept exactly same to the IM prepared from these two parent compounds. The only difference between the mixture (0.5 NBA_2_SO_4_:1 NaIR820) and the IM ([NBA][IR820]) is the presence of spectator ions in the mixture. These spectator ions were removed in the IM during its synthesis (as mentioned in Equation (4)).

**Figure 2.**
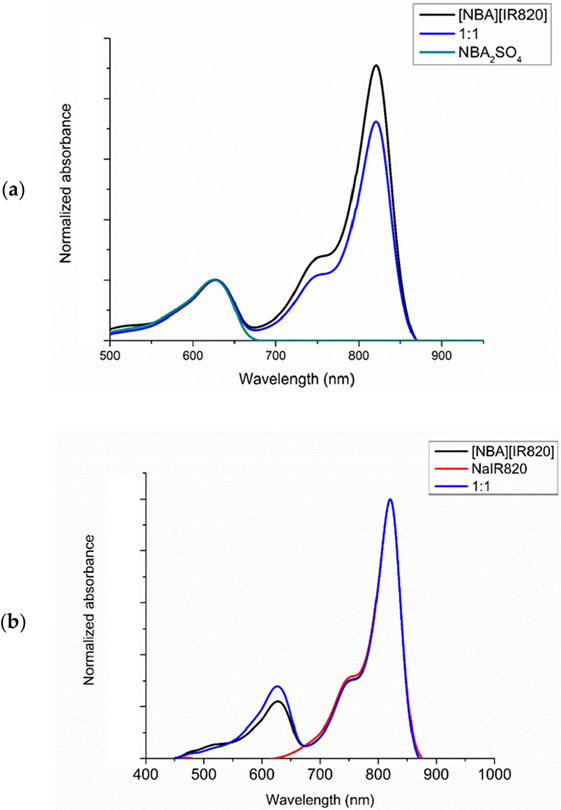
Absorption spectra normalized (**a**) at 629 nm and (**b**) at 820 nm in ethanol.

The NBA_2_SO_4_ exhibits an absorption wavelength maximum at 629 nm and the NaIR820 parent compound displays the absorption wavelength maxima at 820 nm with a shoulder around 755 nm. When the counterion (Na^+^) of IR820^−^ is replaced with NBA^+^ to form [NBA][IR820] IM, a broad absorption spectrum is observed due to the presence of both ionic dyes, which absorb at different wavelengths of electromagnetic radiation, as depicted in Figure S4. A similar shape is observed in the mixture of the two parent dyes. There is no detectible shift discerned in the shape and absorption wavelength maxima peaks for NBA^+^ and IR820^−^ in the IM as well as in the mixture. It indicates that no structural changes occurred in the molecules during synthesis of IM. However, the absorption peak intensity of the peak is greatly affected. To understand these changes, normalized graphs at 629 nm wavelength (Figure 2a) and 820 nm wavelength (Figure 2b) are plotted. A significant increase in absorption intensity of the IR820^−^ is observed in the IM as compared to the mixture (Figure 2a). In contrast, normalization at 820 nm peak showed reduced absorbance of the IM at 629 nm peak in comparison to the mixture (Figure 2b). To explain these interesting results, further experiments were conducted.

**Figure 3.**
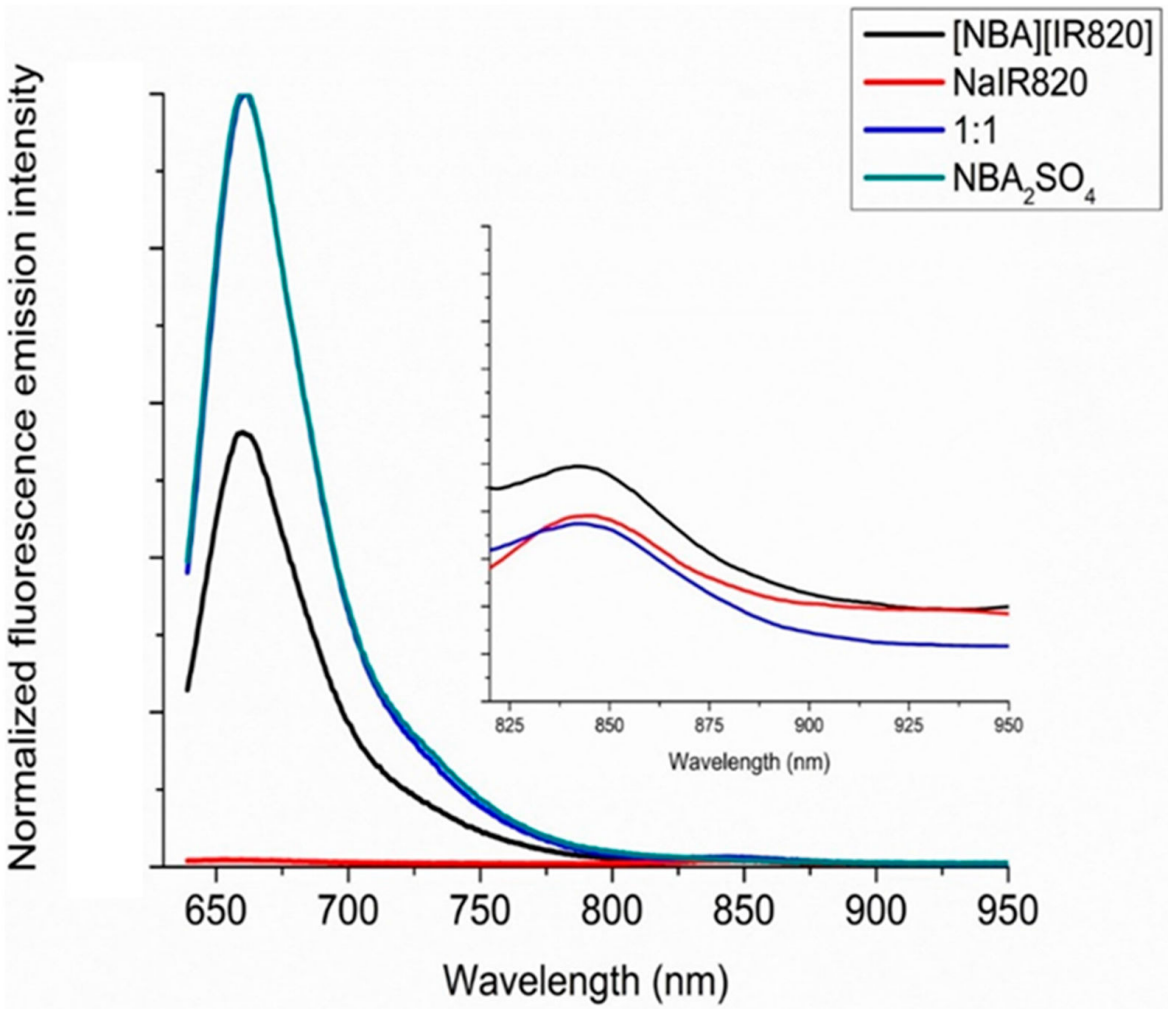
Normalized (with respect to parent NBA_2_SO_4_ at 664 nm) fluorescence emission spectra of NaIR820, NBA_2_SO_4_, the mixture consisting of 0.5 NBA_2_SO_4_:1 NaIR820, and [NBA][IR820] IM at 629 nm excitation wavelength in ethanol.

The fluorescence emission spectra of NBA_2_SO_4_, NaIR820, the mixture, and [NBA][IR820] IM are recorded in ethanol at two different excitation wave lengths, i.e., 629 nm and 820 nm. All the normalized fluorescence emission spectra with respect to parent NBA_2_SO_4_ are presented in Figure 3.

All NBA^+^ containing compounds exhibited the emission wavelength maxima at 664 nm when excited at 629 nm. Additionally, a peak at 845 nm was observed in all IR820^−^-containing compounds. The peak shape and fluorescence emission wavelength maxima did not significantly change in different molecules containing NBA^+^ and IR820^−^ ions. The NaIR820 compound did not show a fluorescence emission peak at 664 nm upon excitation at 629 nm wavelength due to the absence of NBA^+^. However, both the mixture and the IM exhibited the intense emission peak at 664 nm due to the presence of NBA cation. All fluorescence emission graphs were normalized with respect to the NBA_2_SO_4_ at 664 nm. It was examined that the fluorescence emission intensity of NBA^+^ is significantly decreased in the IM in comparison to the parent NBA_2_SO_4_ compound when excited at 629 nm (NBA^+^ excitation wavelength, Figure 3). However, the 1:1 mixture of the dyes did not show any reduction in fluorescence emission intensity regarding the parent NBA_2_SO_4_ compound. Additionally, an increase in emission peak intensity at 845 nm is observed in the IM with respect to the parent NaIR820 dye when excited at 629 nm. The mixture of two dyes did not exhibit any difference in emission intensity with reference to the NaIR820 parent compound. When these compounds were excited at 820 nm (IR820^−^ wavelength maxima), the IM and the mixture exhibited the same intensity (Figure S5). Detailed analysis of absorption and the fluorescence spectra demonstrate that IM photodynamic properties are significantly different than the mixture. The significant changes in the IM such as the increase in IR820^−^ absorbance at 820 nm and decrease in emission intensity of NBA^+^ at 664 nm, as well as the enhanced fluorescence emission intensity of IR820^−^ at 845 nm when excited at 629 nm led us to investigate the FRET phenomenon in the IM.

**Figure 4.**
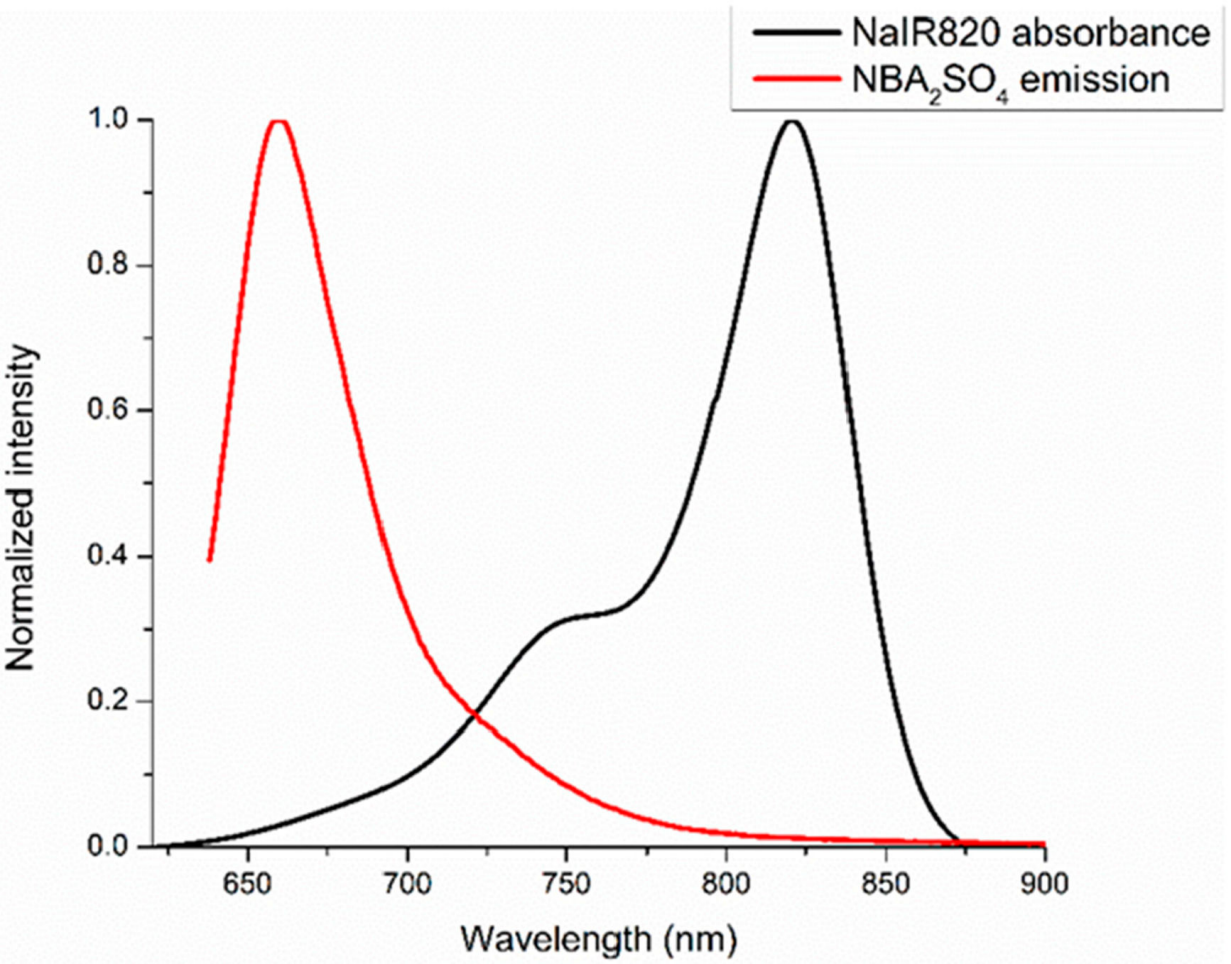
Normalized fluorescence emission spectra of NBA_2_SO_4_ (donor) and absorption spectra of NaIR820 (acceptor) in ethanol.

The wide fluorescence emission spectra of NBA^+^ exhibits overlapping with the absorption spectra of IR820^−^, as displayed in Figure 4. Therefore, it is possible that NBA^+^ can serve as a donor whereas IR820^−^ can act as an acceptor in a FRET compound. Thus, there is a possibility of FRET which caused the reduction in the fluorescence emission intensity of NBA^+^ (donor) as well as the enhanced absorbance and fluorescence emission intensity of IR820^−^ (acceptor) in the IM. The changes in the fluorescence emission intensity for IR820^−^ are only observed when excited at donor (NBA^+^) excitation wavelength (629 nm). As mentioned earlier, the spectral overlap between donor fluorescence emission and acceptor absorption spectra is the primary requirement of FRET. As a result of FRET, the fluorescence emission peak arising from the donor (NBA^+^) should exhibit a decrease in intensity due to the energy being transferred to the acceptor moiety that consequently enhances the fluorescence emission intensity of the acceptor (IR820^−^) in the FRET process [34]. This phenomenon is observed in Figure 3. In addition to spectral overlap, the Förster distance between the donor and acceptor ions is a critical requirement for FRET, as previously stated. It is possible that the presence of the counterions, Na^+^ and SO_4_^2−^, in the mixture of dyes prevents the donor and acceptor ions from being in close proximity. However, the absence of spectator ions in IM provides strong evidence of FRET characteristics.

The fluorescence emission spectra for all compounds are recorded at 820 nm excitation wavelength, as well. The similar peak shape and emission wavelength maxima (845 nm) is observed in IR820^−^-containing dyes (Figure S5). The reason for no change in the fluorescence emission intensity between the mixture and IM at 820 nm excitation wavelength is the lack of energy transfer process. The energy transfer process is only possible when excited at a donor wavelength that augments the acceptor fluorescence emission.

The quantum yield measurements are also performed to further confirm the FRET mechanism in IM. Herein, the absolute quantum yield of the parent donor and acceptor compounds as well as IM are measured using an integrated sphere [35]. All absolute quantum yield values for parent compounds are recorded at their respective excitation wavelengths. The absolute quantum yield acquired for parent compounds (NBA_2_SO_4_ = 27% and NaIR820 = 4.20%) are in agreement with the literature values [36,37]. The IM exhibited donor (NBA^+^) and acceptor (IR820^−^) emission peaks, but the absolute quantum yield values are reported for NBA^+^. The IM quantum yield of the donor was recorded to be 8.04%. Examination of the results revealed that the quantum yield of the IM at 629 nm excitation wavelength decreased significantly relative to the NBA_2_SO_4_ parent compound. Thus, the decrease in donor (NBA^+^) quantum yield further supports the possibility of the FRET phenomenon in the IM.

A detailed examination of all the changes in the photophysical properties indicated that FRET is occurring in IM but not in the mixture of two dyes that are used to develop the IM. The only difference in the [NBA][IR820] IM and the mixture of both dyes (NBA_2_SO_4_ and NaIR820) is the absence and the presence of the counterions Na^+^ and SO_4_^2−^, respectively, thus indicating that the removal of counterions helps to attain the FRET mechanism in the IM, which was not observed in the mixture of parent compounds. As stated earlier, the spectral overlap between the donor’s emission and the acceptor’s absorption spectra is not the only criteria for FRET phenomenon; the optimum distance between the donor and acceptor moieties is also required. In the mixture, the presence of counterions prevents the donor and acceptor moieties to come in close proximity in the solution. For this reason, the mixture does not show any possibility for the FRET mechanism to occur, while the absence of counterions in the IM aids to bring the donor and acceptor ions in close proximity (<10 nm). Previous works have confirmed that the ions in the IMs remain associated in the solution (as ion pairs) by observing conductivity inconsistent with the Nernst–Einstein approximation, indicating that there is a net zero charge for the ion pair [38]. Therefore, energy transfer between the two ionic species in an IM is possible.

### 3.3. FRET Calculations

The quantum yield and other spectral data demonstrated the possibility of FRET in the IM. Therefore, all FRET calculations such as overlap integral, theoretical Förster distance between donor and acceptor, and FRET efficiency are calculated for the IM. The results for all FRET calculations are presented in Table 1. First, we observed the spectral overlap (Figure 4), and the spectral overlap integral between donor NBA_2_SO_4_ dye and acceptor NaIR820 dye was calculated using Equation (1) [26]. The resultant value of spectral overlap reported in Table 1 indicates moderate overlap between donor and acceptor ions in IM. Nevertheless, it can be improved by varying the ions, which shows better spectral overlap to develop FRET-based IMs. The beauty of IM is the simplicity of tuning the properties in a compound by using task-specific ions. Their simple synthesis makes them ideal to develop more FRET-based IMs in the future for a specific application.

Next, the Förster distance in the IM was also calculated to prove the reasoning of the FRET process in the IM in contrast with the mixture of two dyes. For an efficient energy transfer, NBA^+^ and IR820^−^ photosensitizers must be within 10 nm of each other. To calculate this theoretical Förster distance (*R*_0_), Equation (2) is employed. The resulting value proves that donor and acceptor ions are present in close proximity in the IM.

These calculated values confirmed the FRET mechanism in the IM. The IM exhibits the possibility of the FRET mechanism when there is significant spectral overlap between two ions present, which is verified by the spectral overlap integral value (Table 1). The spectral overlap is also possible in the mixture. However, the presence of counterions in the mixture hindered the dyes from acquiring the minimum distance required for energy transfer to occur. Therefore, the FRET mechanism was not observed in the photophysical characterization results for the mixture. Thus, removing the spectator counterions is crucial to bringing the ions in close enough proximity to acquire FRET in an IM. One cannot obtain the FRET mechanism in an ionic compound just by mixing a donor compound with an acceptor compound. The removal of counterions is the key parameter to attain FRET characteristics in an ionic compound.

Finally, the FRET efficiency was calculated between donor and acceptor ions in the IM. For that, Equation (3) was employed. To determine FRET efficiency, the integrated fluorescence emission of the NBA_2_SO_4_ parent compound was compared to the integrated fluorescence emission of the [NBA][IR820] IM. The fluorescence spectra of NBA^+^ in absence (NBA_2_SO_4_) and presence of the IR820^−^ ([NBA][IR820]) are displayed in Figure 3. The decreased fluorescence emission intensity of NBA^+^ in [NBA][IR820] IM in comparison to the parent NBA_2_SO_4_ fluorescence emission revealed that energy is transferred from NBA^+^ to IR820^−^ in the IM [39]. When the mixture of two dyes is compared to the NBA_2_SO_4_ parent compound, there is no observable decrease in fluorescence emission intensity. This concludes that no energy transfer is occurring in the mixture. The present FRET efficiency is tabulated in Table 1, along with the theoretical Förster distance and spectral overlap integral. All the resulting values for overlap integral and Förster distance between two ionic moieties supports the FRET in the IM. Nevertheless, the FRET efficiency is almost 32%, due to minimum spectral overlap between donor and acceptor ions, as observed in Figure 4 and obtained from Equation (1). The FRET efficiency value can be improved further by maximizing spectral overlap between the two ionic moieties in an IM that serves as a donor and an acceptor. The results obtained for the first IM-based FRET compound demonstrate that the FRET process can be achieved using a simple synthetic ion exchange approach. This class of IMs can be exploited to develop other FRET compounds for a variety of applications by selecting task-specific ions in the IM synthesis. To the best of our knowledge, this is the first study that reports that an IM can be designed to acquire FRET characteristics using a very facile, prompt, and cost-effective synthesis approach.

### 3.4. Temperature Studies

Since DSSCs are exposed to sun, it is easy to see the temperature change in the solar cells. The FRET phenomenon in IM improves the spectral properties of the dye in DSSCs. However, it is very important to investigate the temperature-dependent FRET variables. Therefore, absorbance and fluorescence emission spectra of all samples were recorded at different temperatures. The results demonstrated minimal changes in intensity for the parent dyes and the IM at different temperature, as displayed in Figures S6 and S7. This finding also validates the thermal stability acquired from TGA (Figure S3), where all samples showed little degradation around ~290 °C. The insignificant degradation at an elevated temperature and stable photophysical properties demonstrate the suitability of organic dye-based IM for high-temperature applications.

As for the effect of temperature on the FRET parameters, minor changes in the FRET parameters values were observed from room temperature to 70 °C (Table 2). The spectral overlap integral values are in the same order of magnitude at different temperatures. Similarly, the *R*_0_ values are very consistent. In contrast to the Förster distance and spectral overlap integral, the FRET efficiencies on the other hand are more sporadic. The maximum FRET efficiency is attained at 50 °C while at other temperatures, these FRET efficiency values are relatively similar.

## 4. Conclusions

This study presents, for the first time, a new class of compound, IM, that exhibits FRET characteristics. The IM approach to developing a compound with FRET characteristics is very simple, rapid, and economical. Furthermore, the tunable characteristics of IMs make them ideal to develop other FRET-based compounds for a variety of applications. The key to obtaining the FRET mechanism in the IM but not in the mixture is proved by comparing the photophysical properties of the IM and the mixture of two parent dyes. The absence of spectator ions is essential to attain the FRET mechanism in the ionic compounds. All FRET parameters are calculated at different temperatures, which shows that IMs exhibit very stable photophysical properties at elevated temperatures and are thermally stable. For the first time, the concept of FRET is introduced in an IM by pairing an NBA cation with an IR820 anion. All required parameters demonstrate the FRET characteristics of [NBA][IR820] IM. In the future, the simple strategy to design FRET-based IM with enhanced efficiency can further be improved by selecting different ions with greater spectral overlap.

## Supplementary Material

Supplementary Material Figure S1: Mass Spectra of [NBA][IR820]
Figure S2: NMR spectra of [NBA][IR820]
Figure S3: Thermal stability curve for [NBA][IR820], NaIR820, NBA_2_SO_4_
Figure S4: Absorbance spectra of NBA_2_SO_4_ and NaIR820 parent dyes, [NBA][IR820], and 1:1 mixture of dye content from parent compounds (0.5 NBA_2_SO_4_:1 NaIR820) in ethanol
Figure S5: Fluorescence emission spectra at 820 nm excitation wavelength
Figure S6 Absorbance spectra of NBA_2_SO_4_ (a), [NBA][IR820] (b), and NaIR820 (c) at various temperatures
Figure S7 Fluorescence emission spectra of NBA_2_SO_4_ excited at 629 nm (a), [NBA][IR820] excited at 629 nm (b), [NBA][IR820] excited at 820 nm (c), and NaIR820 excited at 820 nm (d) at various temperatures

## Figures and Tables

**Table 1.** FRET parameters: FRET efficiency, theoretical Förster distance, and spectral overlap integral.

% FRET efficiency	31.20%
Förster distance (*R*_0_, nm)	5.60
Spectral Overlap Integral (*J*(*λ*)/M^−1^cm^−1^nm^4^)	6.83 × 10^15^

**Table 2.** FRET parameters: FRET efficiency, theoretical Förster distance, and spectral overlap integral for a range of temperatures.

	25 °C	40 °C	50 °C	60 °C	70 °C
**% FRET efficiency**	28.82	26.19	36.16	24.04	20.58
**Förster distance (*R*_0_, nm)**	5.64	5.62	5.62	5.63	5.82
**Spectral Overlap Integral (*J*(*λ*)/M^−1^cm^−1^nm^4^)**	6.97 × 10^15^	6.78 × 10^15^	6.76 × 10^15^	6.83 × 10^15^	8.39 × 10^15^
