# Supplementary material for: Understanding of Förster Resonance Energy Transfer (FRET) in Ionic Materials
Source: Sustain Chem. Author manuscript; Available in PMC 2022 Mar 28. (PMC8958797; doi:10.3390/suschem2040031)
Supplement: Supplementary Material — Figure S1: Mass Spectra of [NBA][IR820] Figure S2: NMR spectra of [NBA][IR820] Figure S3: Thermal stability curve for [NBA][IR820], NaIR820, NBA2SO4 Figure S4: Absorbance spectra of NBA2SO4 and NaIR820 parent dyes, [NBA][IR820], and 1:1 mixture of dye content from parent compounds (0.5 NBA2SO4:1 NaIR820) in ethanol Figure S5: Fluorescence emission spectra at 820 nm excitation wavelength Figure S6 Absorbance spectra of NBA2SO4 (a), [NBA][IR820] (b), and NaIR820 (c) at various temperatures Figure S7 Fluorescence emission spectra of NBA2SO4 excited at 629 nm (a), [NBA][IR820] excited at 629 nm (b), [NBA][IR820] excited at 820 nm (c), and NaIR820 excited at 820 nm (d) at various temperatures [file NIHMS1787398-supplement-Supplementary_Material.zip › suschem-1355373-supplementary.pdf]

Supporting information

Understanding of Förster Resonance Energy Transfer (FRET) in Ionic Materials

Amanda Jalihal, Thuy Le, Samantha Macchi, Mujeebat Bashiru, Hannah Krehbiel, Noureen Siraj

Department of Chemistry, University of Arkansas at Little Rock, 2801 S. University Ave., Little Rock AR 72204, USA; anjarman@ualr.edu, ttle@ualr.edu, spmacchi@uarl.edu, mobashiru@ualr.edu, hrkrehbiel@ualr.edu, nxsiraj@ualr.edu

* Correspondence: nxsiraj@ualr.edu; Tel.: (501-916-6544)

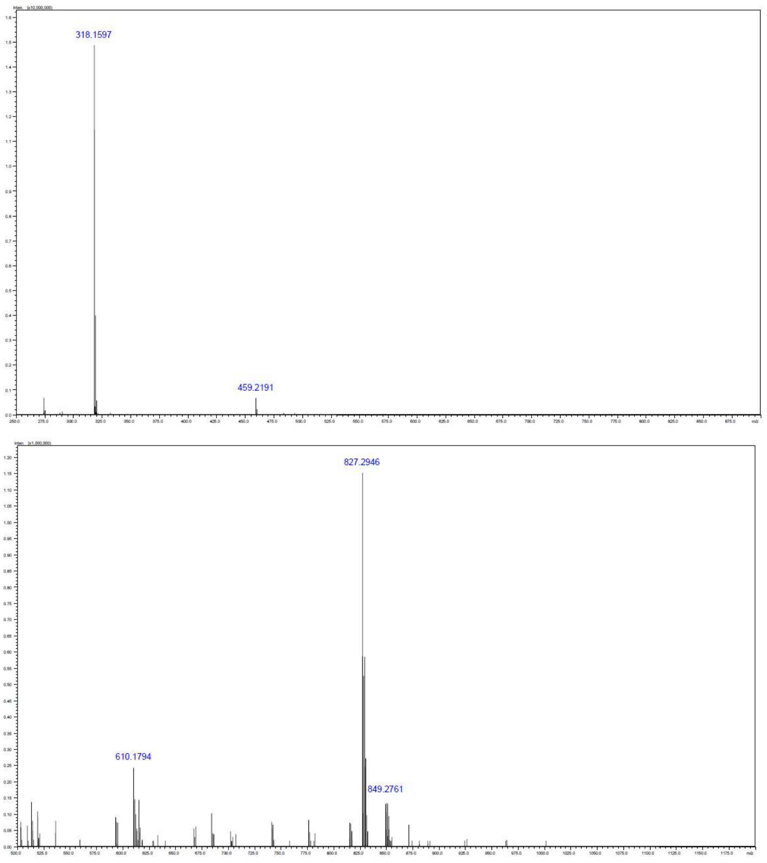

Figure S1. Mass spectra of [NBA][IR820]

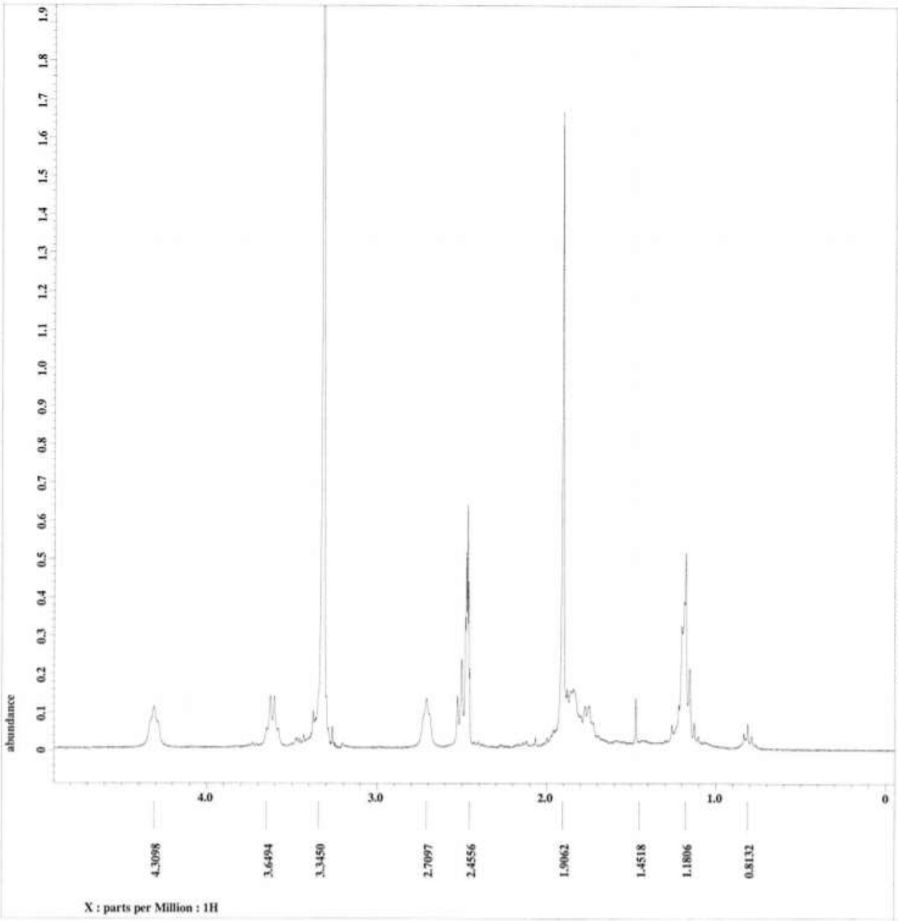

JEOL

```
Filename          = AJ072821a-3.jdf
Author            = NMR-Desrochers
Experiment        = single_pulse.ex2
Sample_id         = DMSO
Solvent           = DMSO-D6
Creation_time     = 28-JUL-2021 11:52:38
Revision_time     = 28-JUL-2021 13:51:26
Current_time      = 28-JUL-2021 13:52:16

Comment           = NBAIR
Data_format       = 1D COMPLEX
Dim_size          = 13107
Dim_title         = 1H
Dim_units         = [ppm]
Dimensions        = X
Site              = ECX 300
Spectrometer      = DELTA2_NMR

Field_strength    = 7.0586013[T] (300[MHz
X_acq_duration    = 2.90717696[s]
X_domain          = 1H
X_freq            = 300.52965592[MHz]
X_offset          = 5[ppm]
X_points          = 16384
X_prescans        = 1
X_resolution      = 0.34397631[Hz]
X_sweep           = 5.63570784[kHz]
Irr_domain        = 1H
Irr_freq          = 300.52965592[MHz]
Irr_offset        = 5[ppm]
Tri_domain        = 1H
Tri_freq          = 300.52965592[MHz]
Tri_offset        = 5[ppm]
Clipped           = FALSE
Mod_return        = 1
Scans             = 8
Total_scans       = 8

X_90_width        = 10.6[us]
X_acq_time        = 2.90717696[s]
X_angle           = 45[deg]
X_atn             = 3[dB]
X_pulse           = 5.3[us]
Irr_mode          = Off
Tri_mode          = Off
Dante_presat      = FALSE
Initial_wait      = 1[s]
Recvr_gain        = 44
Relaxation_delay  = 5[s]
Repetition_time   = 7.90717696[s]
Temp_get          = 22.2[dC]
```

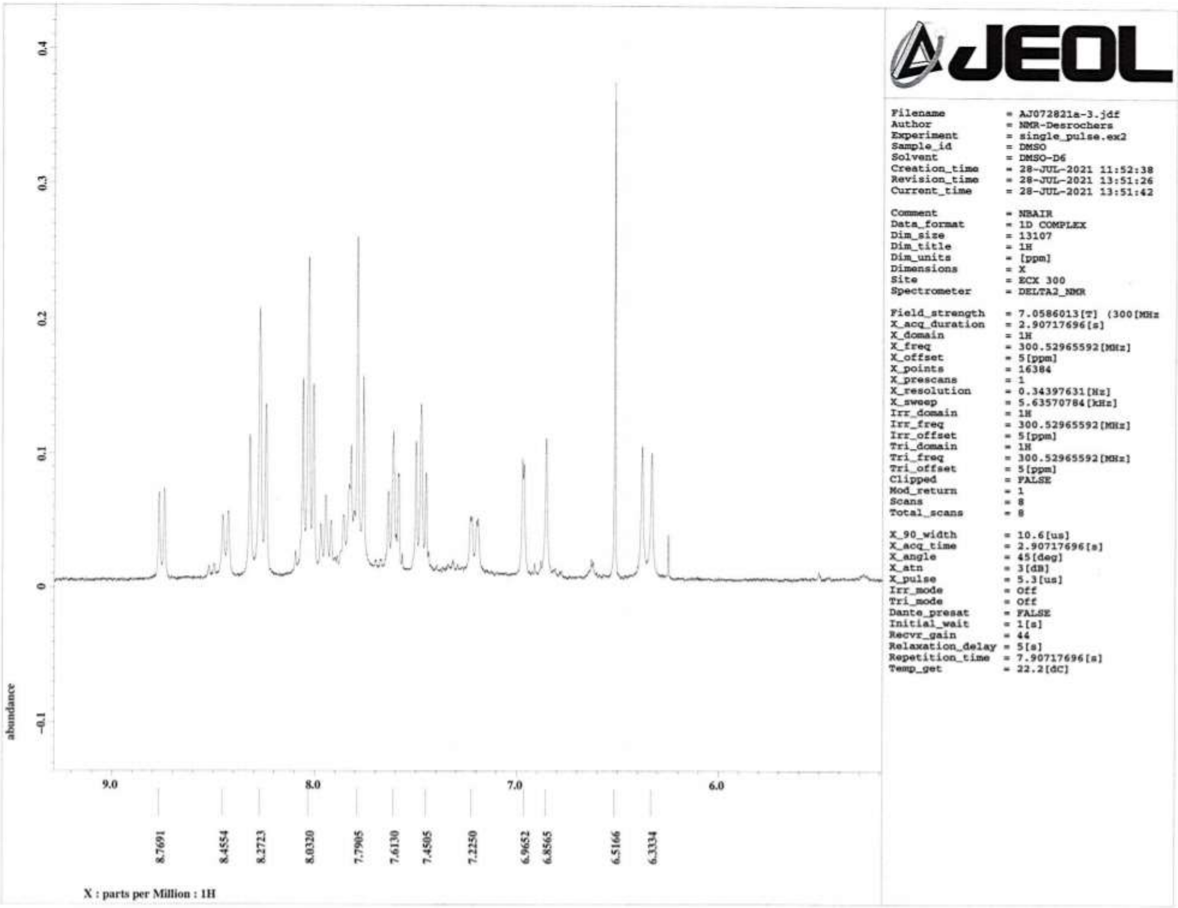

Figure S2. ^{1}H-NMR spectra of [NBA][IR820]: the signal for six protons at δ ^{1}H=1.2 ppm corresponding to identical ethyl groups and δ ^{1}H=7.61 ppm with the proton between the oxygen and cationic nitrogen in NBA^{+}. As for the $IR820^{-}$ moiety, the signal for 22 protons at δ ^{1}H=1.90 ppm, four protons at δ ^{1}H=2.46 ppm corresponding to the protons adjacent to the SO_3^{-} groups, and two protons at δ ^{1}H=4.31 ppm corresponding to the protons next to the cationic nitrogen.

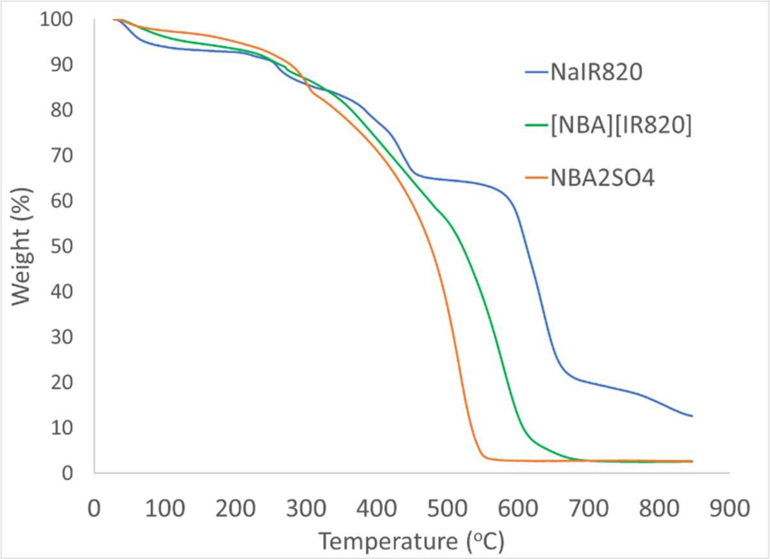

Figure S3. Thermal stability curve for [NBA][IR820], NaIR820, and NBA_2SO_4

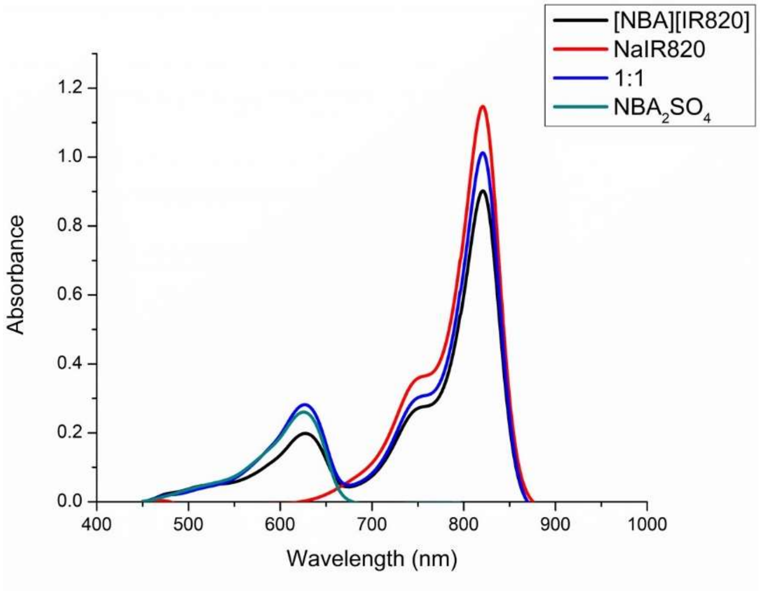

Figure S4. Absorbance spectra of NBA_2SO_4 and NaIR820 parent dyes, [NBA][IR820] IM, and 1:1 mixture of dye content from parent compounds (0.5 NBA_2SO_4: 1 NaIR820) in ethanol. 4 µM concentrations of NaIR820 and [NBA][IR820] were used while 2 µM concentration was utilized for NBA_2SO_4 in absorption measurement. The mixture was held at the same ratio.

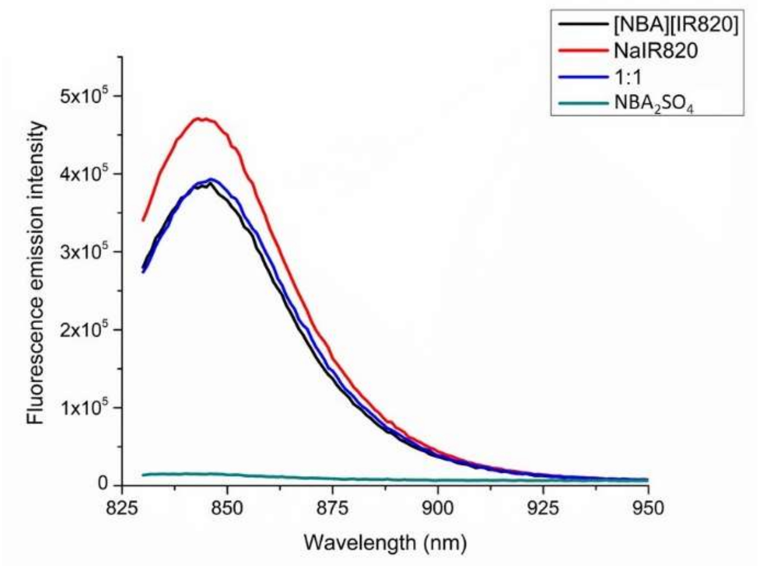

Figure S5. Fluorescence emission spectra at 820 nm excitation wavelength

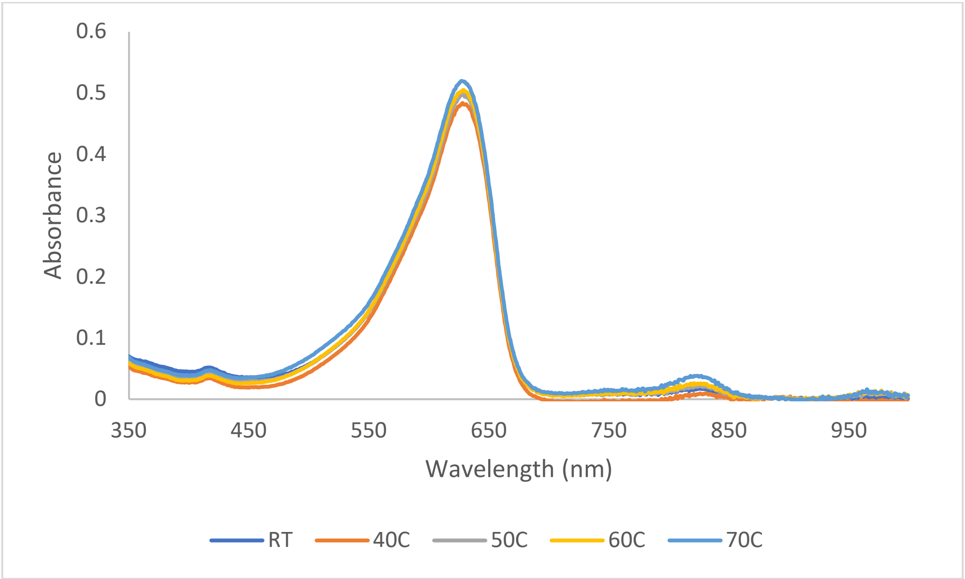

(a)

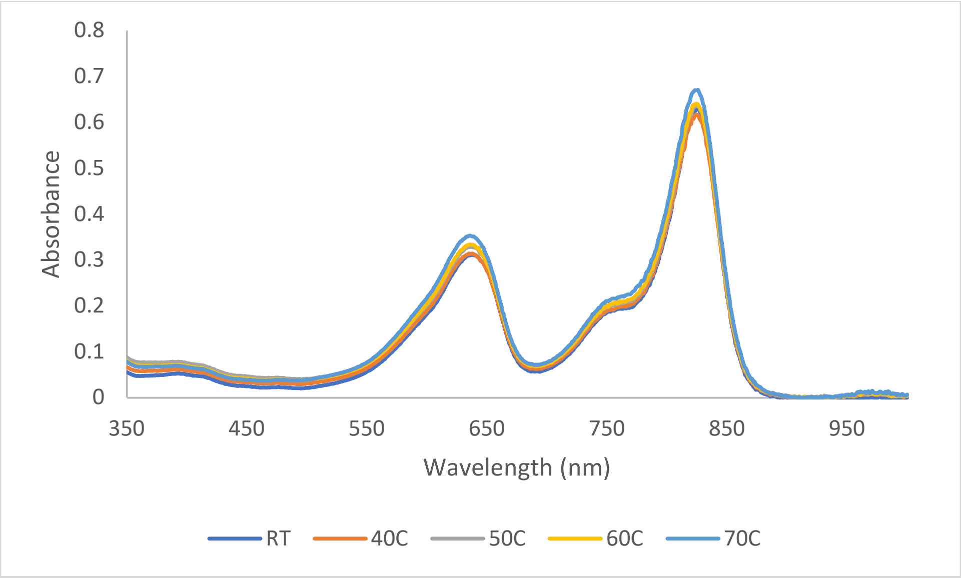

(b)

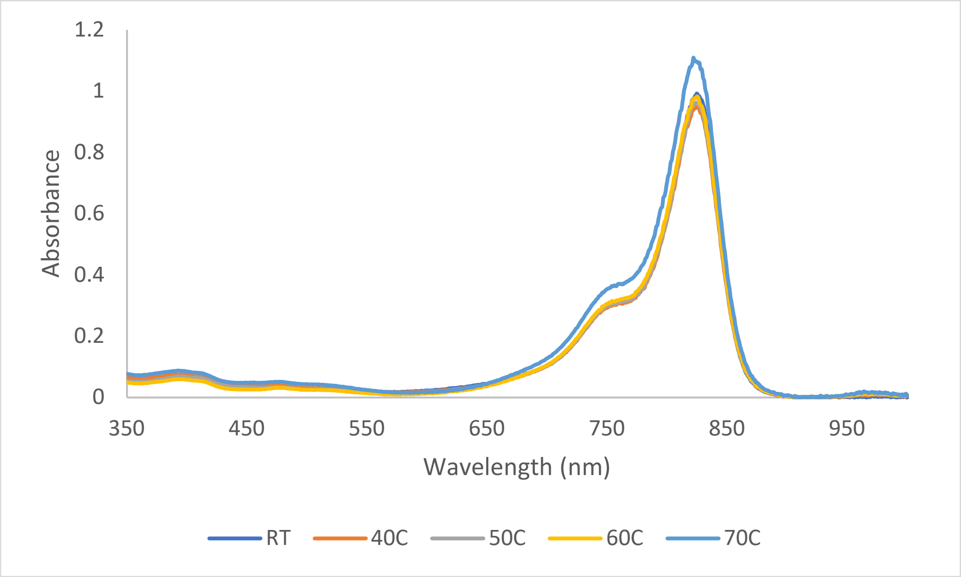

(c)

Figure S6. Absorbance spectra of NBA_2SO_4 (a), [NBA][IR820] (b), and NaIR820 (c) at various temperatures.

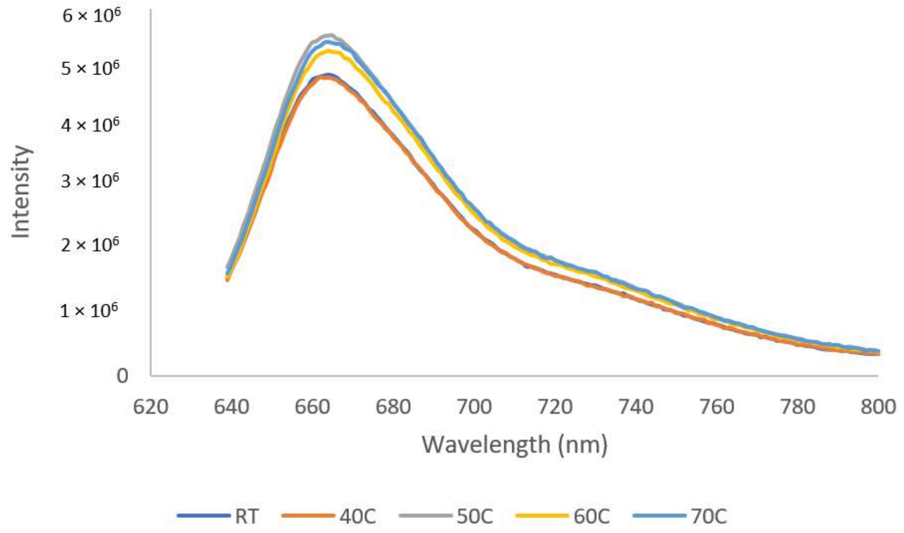
6 × 10^6
5 × 10^6
4 × 10^6
3 × 10^6
2 × 10^6
1 × 10^6
0
Intensity
620 640 660 680 700 720 740 760 780 800
Wavelength (nm)
RT 40C 50C 60C 70C

(a)

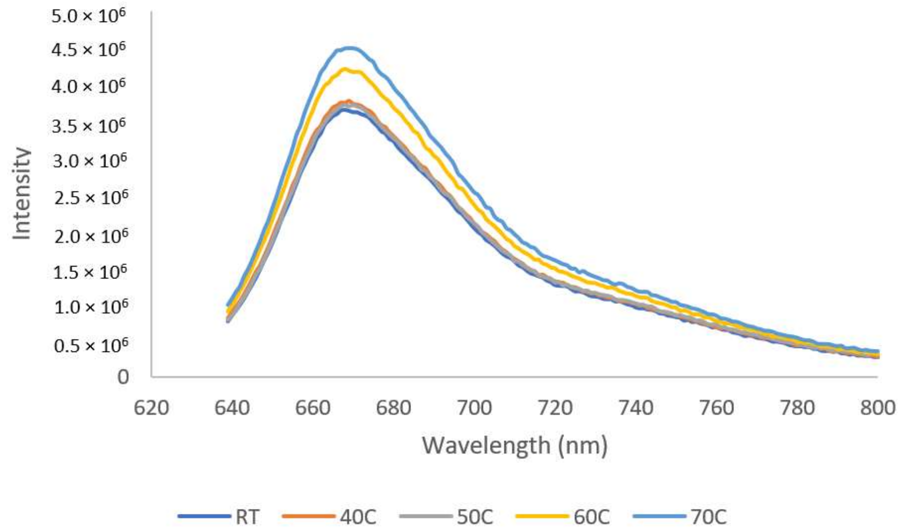
5.0 × 10^6
4.5 × 10^6
4.0 × 10^6
3.5 × 10^6
3.0 × 10^6
2.5 × 10^6
2.0 × 10^6
1.5 × 10^6
1.0 × 10^6
0.5 × 10^6
0
Intensity
620 640 660 680 700 720 740 760 780 800
Wavelength (nm)
RT 40C 50C 60C 70C

(b)

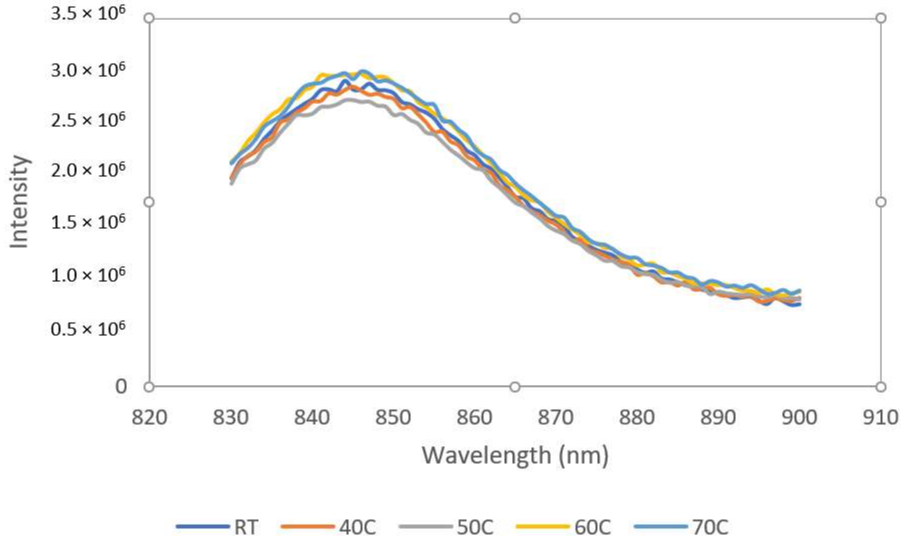
3.5 × 10^6
3.0 × 10^6
2.5 × 10^6
2.0 × 10^6
1.5 × 10^6
1.0 × 10^6
0.5 × 10^6
0
Intensity
820 830 840 850 860 870 880 890 900 910
Wavelength (nm)
RT 40C 50C 60C 70C

(c)

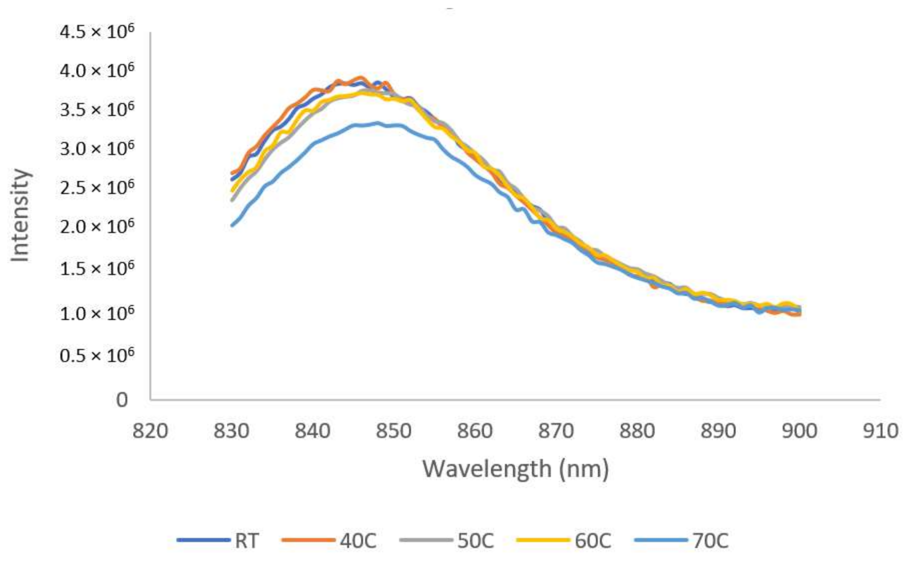

(d)

Figure S7. Fluorescence emission spectra of NBA_2SO_4 excited at 629 nm (a), [NBA][IR820] excited at 629 nm (b), [NBA][IR820] excited at 820 nm (c), and NaIR820 excited at 820 nm (d) at various temperatures.
